# Buckwheat in Tissue Culture Research: Current Status and Future Perspectives

**DOI:** 10.3390/ijms23042298

**Published:** 2022-02-18

**Authors:** Alicja Tomasiak, Meiliang Zhou, Alexander Betekhtin

**Affiliations:** 1 Institute of Biology, Biotechnology and Environmental Protection, Faculty of Natural Sciences, University of Silesia in Katowice, 28 Jagiellonska St., 40-032 Katowice, Poland; alicja.tomasiak@us.edu.pl; 2 Institute of Crop Sciences, Chinese Academy of Agricultural Sciences, Room 405, National Crop Genebank Building, Zhongguancun South Street No. 12, Haidian District, Beijing 100081, China; zhoumeiliang@caas.cn

**Keywords:** common buckwheat, in vitro callus induction, in vitro plantlet regeneration, Tartary buckwheat, tissue culture

## Abstract

Buckwheat is a member of a genus of 23 species, where the two most common species are *Fagopyrum esculentum* (common buckwheat) and *Fagopyrum tataricum* (Tartary buckwheat). This pseudocereal is a source of micro and macro nutrients, such as gluten-free proteins and amino acids, fatty acids, bioactive compounds, dietary fibre, fagopyrins, vitamins and minerals. It is gaining increasing attention due to its health-promoting properties. Buckwheat is widely susceptible to in vitro conditions which are used to study plantlet regeneration, callus induction, organogenesis, somatic embryogenesis, and the synthesis of phenolic compounds. This review summarises the development of buckwheat in in vitro culture and describes protocols for the regeneration of plantlets from various explants and differing concentrations of plant growth regulators. It also describes callus induction protocols as well as the role of calli in plantlet regeneration. Protocols for establishing hairy root cultures with the use of *Agrobacterium rhizogens* are useful in the synthesis of secondary metabolites, as well as protocols used for transgenic plants. The review also focuses on the future prospects of buckwheat in tissue culture and the challenges researchers are addressing.

## 1. Introduction

Plant tissue culture is considered to be an indispensable tool for plant regeneration, rapid multiplication, and propagation of material that is free of pathogens and disease, which characterises a plant with a better response to abiotic and biotic stresses and increased content of desired traits and characteristics [1]. It is, among others, used for research on organogenesis, direct and indirect somatic embryogenesis, callogenesis, hairy root formation, pathways of secondary metabolite production, genetic transformation, protoplast fusion, somatic hybridisation, and haploid plant production [2]. Tissue culture allows for the production of high throughput homogenous material in a short period [1,3]. Tissue culture aseptic conditions can be adjusted to obtain the desired results. These include medium pH, nutrient supply, hormone supply or lack thereof, optimal temperature and photoperiod. Plant tissue culture is widely used in studies on nutritionally and medicinally valuable plants [4,5]. In vitro culture revolves around the characteristics of cells called totipotency. Totipotent cells are considered to have the ability to regenerate into a new plant and express the full genome. However, it must be emphasised that the cell becomes totipotent under two conditions: its initiation has to be as a single cell and it must proceed autonomously as a single process [6,7]. Fehér [8] argued that, since plant regeneration needs to be induced through the process of organogenesis or somatic embryogenesis (SE) and callogenesis, cells regain totipotency, but have not always been totipotent. Fehér [8] describes the SE process as one where a single embryogenic cell is able to regenerate into a viable plantlet; therefore, this cell is totipotent. However, Fehér [8] also points out that if all plant cells were totipotent, each plant cell would be capable of producing somatic embryos. Moreover, the same author highlights that somatic embryo formation does not always require dedifferentiated or totipotent cells during, for example, callo- and organogenesis where an embryo is initiated from procambial cells [8,9]. Regardless of the ambiguity surrounding the term “totipotency”, the principle of regeneration stays the same. It involves the adjustment of plant growth regulators (PGRs) and culture conditions to obtain the plant material with certain characteristics. PGRs are essential in order to determine the developmental pathway of cells in tissue culture. In vivo, plant calli form in response to wounding or pathogen infection, which further prompts changes in the regulation of PGRs, mainly auxin and cytokinin [10]. Fast-proliferating callus tissue is formed from the cambium cells; however, if the wound is not adjoined to cambium, live cells surrounding it undergo dedifferentiation, become meristematic and proliferate, resulting in callus formation [11]. A callus is a tissue with specific characteristics. It can form from one differentiated cell, and it is classified on the basis of the regeneration capacity. Under certain conditions, callus cells become pluripotent, which can lead to plantlet regeneration [6]. In vitro culture is a stressful environment for the plant. Artificial callus induction is caused in response to biotic and abiotic stresses and is affected by explants, media and PGRs [12]. Exogenous application of these hormones results in the induction of various processes. Increased concentration of PGRs result in the induction of various processes, i.e., increased concentration of auxin results in root formation, whereas a high concentration of cytokinin promotes the regeneration of shoots [1]. The balance between auxin and cytokinin govern callus states of dedifferentiation and differentiation. Adjusted concentrations of these hormones result in the induction of calli from explants under in vitro conditions [3]. In vitro callus induction provides an opportunity to extend the research of plant regeneration, cell totipotency, micropropagation and transgenics. Calli can be induced from various plant parts; reports describe induction from hypocotyls [13,14], young seedlings, cotyledon segments [15], shoots [16], immature inflorescence [17], anthers [18], and protoplasts [19,20].

It has been reported that callus induction involves transcriptional or post-transcriptional regulatory elements, which in turn result in global changes in the expression of genes and translation of proteins [21]. Some varieties of callus give rise to clones with inheritable traits different to those of parent plants. This is due to somaclonal variation, which is affected by explant source, age of parent plant, genotype and protocol for tissue culture [22].

Buckwheat belongs to a genus of 23 species, with the two most commonly cultivated species being *Fagopyrum esculentum* Moench (common buckwheat) and *Fagopyrum tataricum* Gaertn. (Tartary buckwheat) [23,24]. Common buckwheat is endemic to northern China and was subsequently introduced to Russia and Europe. The largest buckwheat producers are Russia, China, France, Poland and Ukraine, but it is also cultivated in North and South America [10,25,26]. It is a minor crop; however, the popularity of it is increasing among consumers because of the presence of gluten-free proteins and amino acids such as lysine, tryptophan, methionine, fatty acids, iminosugars, bioactive compounds such as rutin, orientin, quercetin, vixetin, isoorientin, and dietary fibre, fagopyrins, resistant starch, vitamin A, B-complex vitamins, zinc, sodium, copper, iron and other elements [11]. It has been proven that buckwheat has anti-oxidative, anti-tumour, anti-inflammatory, and anti-fungal, cardio-protective, hepato-protective, neuroprotective, anti-hypertension and anti-diabetic properties. Moreover, it can have cholesterol-lowering effects and improve cognition activities [11,27]. Tartary buckwheat is indigenous to west China and is grown in Bhutan, Nepal and northern part of India [28,29]. In Europe, Tartary buckwheat is cultivated in Luxemburg, Belgium, Germany, Italy and Slovenia [29,30]. It characterises with resistance to frost or drought, plant diseases, UV-B radiation damage and pests when compared with common buckwheat [28]. Moreover, Tartary buckwheat is characterised by over 100-fold higher content of rutin than common buckwheat [31]. It also contains resistance starch, balanced amino acids, increased levels of phenolics and was shown to alleviate symptoms of chronic diseases such as cardiovascular diseases, obesity, hypertension and can reduce intolerance of insulin and glucose in humans [28,32].

Buckwheat is widely susceptible to in vitro conditions which have been researched since 1974 [13] and have since been used to study plantlet regeneration from various explants, calli of different morphogenic capacity induction, organogenesis, somatic embryogenesis and synthesis of secondary metabolites such as phenolic compounds [33,34,35]. This review describes the research and progress of in vitro buckwheat cultures. It summarises reports on plant regeneration, callus induction, and protocols, as well as optimal physical, nutritional and hormonal conditions for buckwheat plant tissue culture. Additionally, it summarises the most recent studies on genetic transformation, hairy root cultures (HRCs), the efficacy of the process and optimal conditions and future prospects for buckwheat tissue culture.

## 2. Buckwheat Tissue Culture

### 2.1. Callus Induction

Plant tissue culture, also termed in vitro culture, is a widely used tool in agriculture and horticulture with different applications. The main advantage of the in vitro propagated material is the high yield of regenerants obtained in a short time. Regenerated plant populations are genetically homogenous, essentially disease-free and can possess desired traits such as low temperature, salinity and drought resistance [1]. Plant tissue culture is also important in terms of the germplasm preservation and imposing strategies for genetic interference and transformation as well as studying processes such as totipotency, differentiation and dedifferentiation and organogenesis [1]. As described by Ikeuchi et al. [36], the formation of a callus is a stage in plant regeneration and exhibits high and efficient regeneration potential.

Buckwheat characterises with high regeneration potential from calli [21]. Several studies have been conducted regarding the callus induction from various explants [13,14,15,18,20,35,37,38]. Table 1 summarises the optimal concentrations of hormones to induce calli from hypocotyls, cotyledons and immature embryos. Yamane [13] was the first to report callus induction from cotyledons and hypocotyls of common buckwheat using 10.0 mg/L of 2,4-dichlorophenoxyacetic acid (2,4-D). Takahata and Jumonji [14] conducted experiments with different concentrations of 2,4-D and 6-Benzylaminopurine (6-BA) and noticed that callus was induced with 6-BA, without the addition of 2,4-D. This is in contrast to work carried out by Yamane [13]. Rajbhandari et al. [16] noted improved callus induction in stem explants compared to leaf explants. Their experiments also showed that the supplementation of leaf and stem explants with naphthalene acetic acid (NAA) and indole acetic acid (IAA) at the concentrations of 0.5, 1.0 and 1.5 mg/L did not result in callus formation [16].

Callus differentiation from hypocotyl-derived protoplasts was the most optimal with 1.0 mg/L 2,4-D and 0.1 mg/L 6-BA [19]. Adachi et al. [19] induced callus formation from hypocotyl-derived protoplasts on MS medium with 1.0 mg/L NAA and 1.0 mg/L 6-BA. Hao et al. [38] described the optimal conditions for callus induction from cotyledons and hypocotyls; it was noticed that a high level of sucrose had an enhancing effect on callus induction and further growth. On the other hand, Woo et al. [40] observed that the sucrose content in the induction of calli had no effect on subsequent somatic embryo development. Interestingly, Gumerova et al. [47], who used the same explants, have achieved optimal callus induction with different concentrations of PGRs. This may depend on buckwheat species specificity or the genotype.

In research on Tartary buckwheat, Rumyantseva et al. [34] used a different basal medium with a number of different vitamins and PGRs, namely 2.0 mg/L thiamine, 1.0 mg/L pyridoxine, 1.0 mg/L nicotinic acid, 2000 mg/L casein hydrolysate, 2.0 mg/L 2,4-D, 0.5 mg/L IAA, 0.5 mg/L NAA, and 0.2 mg/L KT and noted that the callus induction depends on explant species affiliation as well as the stage of immature embryo used during the induction. Wang et al. [45] used MS medium supplemented with 2.0 mg/L 2,4-D and 1.0 mg/L KT and obtained 98.96% induction rate. It is important to highlight that in the literature on buckwheat callus, the terminology is slightly ambiguous. Rumyantseva et al. [34] described four types of calli in common buckwheat: dense globular that proliferates slowly, dense slowly proliferating, loose and rapidly multiplying, and heterogenous slowly proliferating comprising loose globules, and one in Tartary buckwheat: heterogenous slowly growing. The same authors did not observe the loose and dense types; however, it was concluded that the characteristics of the callus during cultivation change, and that the heterogenous callus has the ability to establish calli with loose morphology. The common buckwheat dense-type callus and the heterogenous callus of Tartary buckwheat exhibited the highest organogenic capacity and after the transfer to the regeneration medium it prompted the emergence of stem apices. However, when globular calli (morphogenic) were transferred to the embryonic medium, embryo-like structures were observed after several days [34]. Woo et al. [41] describes somatic organogenesis from leaf explants calli which gave rise to somatic embryos. Park et al. [39] used leaf and stem segments and observed the emergence of proembryogenic/proembryonal complexes (PECCs) on explants and subsequent somatic embryogenesis. This was also observed by Gumerova et al. [33], who noted that the regeneration from PECCs can be achieved in three ways: via the formation of vegetative buds, via the formation of somatic embryos on the PECCs’ surface and via the whole PECC transformation in a sprouting embryo. The term embryogenic callus is also used by Saraswat and Kumar [48], where it was induced from hypocotyls and cotyledons. On the other hand, Valieva et al. [49] describes an embryogenic callus, which can form embryoids, roots and buds, and a morphogenic callus, which can form vegetative buds and roots. They also describe histogenic calli, capable of vascular differentiation, and non-morphogenic callus (NC), which emerges on the surface of the morphogenic callus (MC) after two to three years of culture [49]. It is important to stress that the embryogenic callus is described as the one comprising PECCs and so called ‘soft’ callus by the authors [50]. Research on Tartary buckwheat by Betekhtin et al. [21] describes MC as the one with the ability to undergo somatic embryogenesis, organogenesis and subsequent plant regeneration. It comprises PECCs or pro-embryogenic masses (PEMs) and phenolic containing cells (PCC) on their surface [21]. In the course of callus cyclical development, mature PEMs collapse and new PEMs along with ‘soft’ callus cells (SCC) arise which are elongated and have large vacuoles and starch grains in the cytoplasm [21,34,50,51]. PCCs cover the surface of PEMs and parenchymatous cells localised in the central part of PEMs [21]. Calli exhibiting no apparent regeneration are classified as compact, friable or non-morphogenic calli [52]. The emergence of NC is correlated with metabolic changes of the cells. Calli lose the capability of accumulating and synthesizing pigments such as anthocyanins during a prolonged period of culture [13,21,49,51]. It emerges seldomly from separate foci (once per 30–40 passages) [53]. Tartary buckwheat calli with different morphogenic capacities allow us to conduct comparative analyses of physiological, biochemical and cytological features in long-term cultivated callus lines.

### 2.2. Buckwheat Plant Regeneration

Protocols for buckwheat regeneration from several explants such as hypocotyls [14,20,42,46,47,54,55,56], immature inflorescence [17], nodal segments [57], immature embryos [34,58], anthers [44,59], leaf petioles [60] and cotyledons [40,54,61,62] were developed. In Table 2, the optimal conditions for the plantlet regeneration from different explants, *F. esculentum*, *F. tataricum* and *F. cymosum*, are summarised.

The first description of common buckwheat plantlet regeneration from explants was reported by Yamane [13]. It was reported that plant calli could regenerate plants even after 48 months of culture. Moreover, shoot and root formation was observed in calli transferred to modified LS (Linsmaier and Skoog) medium (supplemented with 15% coconut milk and 3.0 mg/L yeast extract) after 30 to 60 days [13]. Srejovic and Neskovic [15] classified the stages of explants’ growth via organogenesis. Takahata and Jumonji [14] were able to produce regenerants of common buckwheat on MS (Murashige and Skoog) medium with up to 5.0 mg/L 2,4-D and up to 2.0 mg/L, and it was concluded that calli can be induced only with 6-BA, without the addition of 2,4-D. Takahata [17] observed that direct formation of shoots from inflorescence was promoted by supplementation of the media with 0.2 mg/L NAA and 1.0 mg/L of 6-BA. Rajbhandari et al. [16] reported improved plantlet regeneration when MS medium was supplemented with 0.2 mg/L IAA and 2.0 mg/L 6-BA, and found dependency of regeneration on the genotype; however, somatic embryos were not observed. Hao et al. [38] noticed that carbohydrates have a shoot-promoting effect on the cutting site and act as osmotic stabilisers. A similar finding by Lachmann and Adachi [20] reported that the addition of sucrose was important for SE induction in buckwheat. On the contrary, Woo et al. [40] found that increased sucrose content did not have any effect on the callus induction or SE. In terms of callus induction and proliferation, Saraswat and Kumar [48] noticed that the hypocotyl explants responded better in media supplemented with glucose, while cotyledon explants responded better to the medium with sucrose as the carbohydrate source. Park et al. [39] were able to regenerate plants from leaf-derived calli on MS medium devoid of hormones, but the regeneration rate was low and failed to regenerate plantlets from stem-derived calli. Park and Park [62] reported direct organogenesis of multiple shoots from cotyledon explants. Berbec and Doroszewska [63] discovered that LS medium supplemented with TDZ (N-Phenyl-N′-1,2,3-thiadiazol-5-ylurea) is suitable for regeneration only when combined with IAA. When organogenesis was induced from leaf petioles, the optimal conditions were MS medium supplemented with 1.0 mg/L 6-BA, 1.0 mg/L 2iP (6-(γ,γ-Dimethylallylamino) purine) and 1.0 mg/L 2,3,5- TIBA (2,3,5- triiodobenzoic acid). It is speculated that TIBA controls the shoot initiation process by contracting endogenous auxins. This research has also revealed a finding that the rate of seed abortion in regenerated plants is much lower compared to the ones grown directly from seeds [60]. Another investigation was conducted with the use of 7.0 mg/L of silver nitrate (AgNO_3_) and noted improvement in the frequency of shoot regeneration of over 30% [64]. Similar results were obtained by Saraswat and Kumar [48] where the use of AgNO_3_ showed improvements in shoot and root growth. The same authors tested the addition of KNO_3_ to the medium. It was concluded that KNO_3_ does not influence the maturation of somatic embryos, as is reported in SE in other species such as cotton [65]. Saraswat and Kumar [48] speculated that low maturity of somatic embryos supplemented with KNO_3_ is due to the buckwheat being a salt-sensitive crop.

**Table 2:** Optimal conditions for the plantlet regeneration from different explants *F. esculentum*, *F. tataricum* and *F. cymosum*.

Species	Explant	Basal Medium	PGRs	References
*F. esculentum*	Hypocotyl	LS	1.0–10.0 mg/L 2,4-D + 2.0 mg/L NAA	[13]
	Cotyledon	B5	0.1 mg/L 6-BA + 0.1 mg/L IAA	[15]
	Hypocotyl	MS	0.1–0.2 mg/L NAA + 1.0–2.0 mg/L 6-BA	[14]
	Immature embryo	B5	2.2 mg/L 6-BA + 0.17 mg/L IAA + 0.5 mg/L IBA	[58]
	Shoot apex	MS	0.5 mg/L IAA + 2.0 mg/L 6-BA	[66]
	Immature inflorescence	B5	0.2 mg/L NAA + 1.0 mg/L 6-BA	[17]
	Anther	MS	1.0 mg/L 6-BA + 0.2 mg/L IAA	[59]
	Hypocotyl derived protoplast	MS	0.1 mg/L NAA+ 0.5 mg/L 6-BA + 0.1 mg/L GA_3_	[19]
	Leaf and stem	MS	0.2 mg/L IAA + 2.0 mg/L 6-BA	[16]
	Anther	MS	2.5 mg/L 6-BA + 0.5 mg/L IAA	[44]
	Cotyledon	MS + B5 vits	0.5 mg/L IAA + 0.25 mg/L IBA	[61]
	Hypocotyl and cotyledon	LS	0.05–0.1 mg/L TDZ + 0.5 mg/L IAA	[63]
	Leaf and stem	MS	Hormone free	[39]
	Cotyledon	MS	2.0 mg/L 6-BA + 0.2 mg/L KT	[40]
	Nodal segment	MS	1.0 mg/L KT	[67]
	Anther	B5	1.0 mg/I NAA + 1.0–2.0 mg/L 6-BA	[18]
	Hypocotyl	MS	2.0 mg/L 6-BA + 1.0 mg/L KT	[43]
	Hypocotyl	B5	2.23 mg/L 6-BA + 0.17 mg/L IAA	[33,47]
	Leaf	MS	0.2 mg/L KT + 2.0–3.0 mg/L 6-BA	[41]
	Cotyledon and hypocotyl	MS + B5 vits	1.0 mg/L 6-BA	[57]
	Nodal segment and shoot apex	MS + B5 vits	2.0 mg/L 6-BA + 0.2 mg/L IAA	[57]
	Cotyledon	MS	4.0 mg/L 6-BA + 7.0 mg/L AgNO_3_	[68]
	Leaf petiole	MS	1.0 mg/L 6-BA + 1.0 mg/L 2iP + 1.0 mg/L TIBA	[60]
	Hypocotyl	MS	1.0 mg/L KT + 1.0 mg/L 6-BA + 2.0 mg/L IAA	[42]
	Hypocotyl	MS	1.0 mg/L NAA + 1.0 mg/L 6-BA	[55]
	Hypocotyl and cotyledon	MS	0.2 mg/L 6-BA + 0.5 mg/L AgNO_3_	[48]
*F. tataricum*	Immature embryos	MS	0.1 mg/L 6-BA + 0.1 mg/L IAA	[34]
	Hypocotyl derived protoplast	MS	2.0 mg/L NAA + 1.0 mg/L 6-BA	[20]
	Hypocotyl	MS	1.0 mg/L NAA + 0.5 mg/L 6-BA	[55]
	Hypocotyl and cotyledon	MS	3.0 mg/L 6-BA + 1.0 mg/L TDZ	[45]
*F. cymosum*	Immature inflorescence	B5	1.0 mg/L NAA + 1.0 mg/L 6- BA	[17]
	Adventitious buds	MS	2.0 mg/L 6-BA + 0.5 mg /L TDZ + 0.2 mg/L NAA	[69]
	Nodal segments	MS	2.5 mg/L IBA	[5]

Reports on Tartary buckwheat regenerations were first described by Rumyantseva et al. [34] who were able to demonstrate plant regeneration from calli cultured for a period of 18 months. It was noticed that the embryoids developed faster and spontaneous embryogenesis occurred more frequently, compared to common buckwheat. Han et al. [46] reported moderate frequency of Tartary buckwheat plantlet regeneration by supplementing MS medium with 1.0 mg/L IAA, 1.0 mg/L KT, 2.0 mg/L 6-BA and 0.5 mg/L TDZ. However, the authors did not mention the percentage of regenerated plants. Wang et al. [45] reported 56% plantlet regeneration level from callus on the MS medium supplemented with 2.0 mg/L 6-BA and 1.0 mg/L KT. These authors observed the level of shoot induction of 69% on MS basal medium with the addition of 3.0 mg/L 6-BA and 1.0 mg/L TDZ. Induction of roots was obtained on half-strength (½) MS medium with 1.0 mg/L IBA, which resulted in 75% of plantlets surviving after being transferred to soil with field conditions [45]. Advances in buckwheat regeneration protocols can be used as protocol for transformation which in turn will create opportunities to examine metabolic and molecular regulation of significant components such as gluten-free proteins, fatty acids, flavonoids and vitamins. The literature in terms of callus induction in buckwheat focuses only on common and Tartary buckwheat, while it is important to highlight that there are 23 buckwheat species.

### 2.3. Hairy Root Culture

A type of tissue culture which has received increasing attention in recent years is the hairy root cultures (HRCs) [64,70,71,72,73,74,75,76,77,78,79,80,81,82,83]. These are derived from infecting the explants with *Agrobacterium rhizogenes* and occurs via the transfer of the root-inducing (Ri) bacterial plasmid, which in consequence prompts the induction of hairy root (HR) syndrome [84] and results in the abundant growth of neoplastic roots that can be cultured under in vitro conditions [77]. Neoplastic roots are singular due to their biosynthetic and genetic stability, which improves the activity of growth regulators [78]. There are multiple advantages of HRCs, which makes them a valuable system for research on secondary plant metabolites such as flavonoids. Other advantages include high growth rates without the addition of PGRs, and in certain cases, without incubation under light. HRCs are genetically stable and respond well when the conditions of culture such as carbon source and concentration, medium pH, white fluorescent light and temperature are adjusted for optimal secondary metabolite secretion [85]. However, the most prominent feature of HRCs is the fact that *A. rhizogenes* is able to manipulate the host in order to increase the chance of a successful transformation. Thus, the *Agrobacterium*-mediated transformation of HRCs provides an easy and fast way to introduce and express foreign genes in cells that are able to carry out the synthesis of certain secondary metabolites [86]. It has been reported that *A. rhizogenes* strain number 15,834 is one of the most widely used strains for hairy root induction as well as their production of secondary metabolites [76,87,88,89]. As for the production of secondary metabolites in callus cultures of *Fagopyrum* species, it was reported by Moumou and Trotin [90] that the flavonoid content was relatively low in calli grown in darkness compared to ones grown under the light. Nonetheless, secondary metabolite content was significant, especially the content of two dimeric proanthocyanidins: B2 and B2-3′-O-gallate and two catechins: epicatechin and epicatechin 3-O-gallate [90,91,92]. Therefore, as a source of bioactive secondary metabolites such as flavonoids, transgenic technology involving *Fagopyrum* species, especially through HRCs is important in the investigation of their molecular and metabolic regulation and genetic engineering. In Table 3, the optimal conditions for the production of bioactive compounds with *A. rhizogenes* from different explants, *F. esculentum* and *F. tataricum*, are summarised.

The first reports on HRCs in common buckwheat date to 1990 when Neskovic et al. [93] researched the inoculation of *A. rhizogenes*, which led to the development of hairy roots on stems. Two strains were used, i.e., ATCC 15834 and ATCC 13332, to induce HRCs and obtain the efficiency of HRCs formation at 75.8% and 24.1%, respectively, and overall formation of HRCs from inoculated explants was 39.7%. It was concluded that this species is very responsive to *Agrobacterium* which in the future could be used as a vector in genetic transformation [93]. Moumou and co-workers [91] discovered that selected cultures of calli induced from common buckwheat hypocotyls produced significantly high quantities of flavonoids, procyanidins and catechins in particular, which were not present in original plants. Trotin et al. [94] established HRCs by infecting hypocotyl explants of common buckwheat with *A. rhizogenes* strain ATCC 15834 and observed the synthesis of flavonoids: catechin, epicatechin, epicatechin-3-O-gallate, procyanidin B2 and procyanidin BZ3’-O-gallate. It was concluded that the HRCs exhibited the highest concentration of procyanidin B2-3′-O-gallate, suggesting that HRCs characterise in the preferential synthesis of galloylated derivatives of monomeric and dimeric classes as well as inverted content of catechin and epicatechin when compared to calli, confirming that HRCs contain more flavonoids than regular roots, in vitro normal roots and calli. Polyphenolics were also investigated in HRCs induced by *A. rhizogenes* strain MAFF 03-01724, which induces similar secondary metabolite production in roots compared to those in intact plants [95]. The effect of different media on HRCs was investigated and led to the conclusion that the most suited is MS medium; however, the most promising result was that the HRCs produced ten times more rutin than the field-cultivated plants [95]. About a decade later, Lee et al. [96] used a different strain of *A. rhizogenes* (R1000) and tested the production of rutin in common buckwheat HRCs. It was found that the quantity of rutin produced by HRCs was notably higher when compared with the control. It was also noticed that the addition of auxins (IAA) positively affected rutin production and the most suitable medium for the highest yield of HRCs was ½ MS. Research led by Kim et al. [86] provided a protocol for common buckwheat HRCs transformation via affecting stems with *A. rhizogenes* strain ATCC 15834 containing pB1121 binary vector. Rapidly growing clones were obtained, which produced 2.6 times more rutin than the wild type roots [97]. This work provided the efficient and multifaceted system for investigating the molecular regulation of phenolic compound biosynthesis as well as evaluating the system’s potential for metabolically engineering and rutin production [97]. Park et al. [74] demonstrated that the expression of the transcription factor AtMYB12 resulted in increased levels of rutin in common buckwheat transgenic HRCs. The family of MYB transcription factor is commonly present within higher plants [74]. It is known to participate in diverse biochemical and physiological processes, which include, among others, response to abiotic stress, influencing secondary metabolism pathways and controlling cell morphogenesis [98]. The accumulation of flavonols was observed as a result of the over expression of AtMYB12, which was primarily identified in *Arabidopsis thaliana* [99,100]. As a conclusion of Park et al. [75], it was noted that although AtMYB12 notably affected the production of rutin in transgenic buckwheat HRCs, a more effectively engineered metabolic pathway is required in order to allow the identification of transcription factors from the *Fagopyrum* species [75]. Gabr et al. [70] measured the content of phenolic acids in HRCs of common buckwheat obtained by infecting roots, stems and leaves with *A. rhizogenes* strain A4 and observed three-fold higher content of chlorogenic acid than in the control in all treated explants, with HRCs from roots containing most of the acid. HRCs from roots also exhibited an increased content of p-anisic and caffeic acids. The same authors in 2019 investigated the content of rutin in HRCs of common buckwheat in explants from leaves, stems and roots. It was reported that the rutin content was higher in control root compared to HRCs from stem and leaves, which led to the conclusion that the suppression of rutin accumulation in HRCs is a form of plant defence against stress. It was also concluded that the production of rutin depends on the type of explant, and moreover that HRCs displayed increased levels of histidine, valine, lysine and isoleucine and total content of flavonoids and the antioxidant activity [71].

**Table 3:** Optimal conditions for the production of bioactive compounds with *A. rhizogenes* from different explants *F. esculentum*, *F. tataricum*.

Species	Explant	*A. rhizogenes* Strain	Basal Medium	Bioactive Compounds	References
*F. esculentum*	Stem	ATCC 15834 ATCC 13332	½ B5 (half strength)	-	[93]
	Hypocotyl	ATCC 15834	B5	Catechin, epicatechin, epicatechin-3-O-gallate, procyanidin B2 and procyanidin B2-3′-O-gallate	[94]
	Leaf	MAFF 03-01724	MS	catechin, epicatechin, epicatechin 3-O-gallate procyanidin B-1 and procyanidin B-2 3′-O-gallate	[95]
	Leaf	R1000	MS	Rutin	[96]
	Stem	ATCC 15834	MS	Rutin	[97]
	Stem	R1000	MS	Rutin	[75]
	Leaf, stem and root	A4	MS	Rutin, chlorogenic acid, hyperoside, caffeic acid, p-anisic acid	[70]
	Steam, leaf	A4	MS	Rutin, hesperidine, kaempferol-3-O-rutionoside	[71]
*F. tataricum*	Stem	R1000	MS	Rutin, quercetin, epicatechin, catechin hydrate, gallic acid, ferulic acid, chlorogenic acid, and caffeic acid	[86]
	Stem	R1000	MS	Rutin, epicatechin, gallic acid, epigallocatechin, caffeic acid, catechin hydrate, chlorogenic acid	[74]
	Stem	R1000	½ SH	Rutin, epicatechin, gallic acid, epigallocatechin, caffeic acid, catechin hydrate, chlorogenic acid	[81]
	Hypocotyl	R1000	MS	Rutin, quercetin, gallic acid, caffeic acid, ferulic acid, 4-hydroxybenzoic acid	[79,80]
	Leaf	Ri1601	MS	Rutin and quercetin	[99,100]
	Hypocotyl	R1000	½ MS	Cyanidin 3-O-glucoside, cyanidin 3-O-rutinoside	[64]
	Stem	R1000	½ MS	Rutin, quercetin, chlorogenic acid, 4-hydroxybenzoic acid, caffeic acid, ferulic acid, cyanidin 3-O-glucoside, cyanidin 3-O-rutinoside,	[72]

Tartary buckwheat has over 26-fold higher content of rutin than common buckwheat making it more suitable for researching HRCs and their production of bioactive compounds [101]. Kim et al. [86] quantified rutin, quercetin, epicatechin, catechin hydrate, gallic acid, ferulic acid, chlorogenic acid, and caffeic acid in HRCs of Tartary buckwheat obtained from stems infected with an *A. rhizogenes* R1000 strain. The results showed that rutin and epicatechin in HRCs were 10 times and 5 times higher, respectively, compared with wild-type roots [86]. Park et al. [74] used the same *Agrobacterium* strain as Kim et al. [86] to produce HRCs from Tartary buckwheat stems and observed two morphological phenotypes emerging in their cultures. Approximately 80% of the established HRCs had well-developed primary roots and a few secondary roots, and are therefore termed as a “thick phenotype”, and the remaining 20% displayed traits characteristic of those of primary roots with a profusion of root hairs, which they named the “thin phenotype”. The “thin phenotype” had a higher number of hairy roots as well as higher growth rate than the “thick phenotype”. Park et al. [74] concluded that selecting the optimal morphological phenotype of HRCs is crucial for improved production of secondary metabolites under in vitro conditions. Attempts to optimise the growth and enhance the synthesis of phenolic compounds in HRCs with the basal medium and the addition of PGRs were made with the use of R1000 strain in Tartary buckwheat stems [81]. It was established that the optimal medium for HRC growth and phenolic compounds biosynthesis was half-strength SH (Schenk and Hildebrandt) medium. As for the PGRs, the best results were obtained with 0.5 mg/L indole-3-butyric acid (IBA), which resulted in 24% more growth than in the control [81]. In contrast to these findings, Park et al. [64] showed that high concentration auxins, namely IBA, NAA and 2,4-D had no effect on HRC growth. However, enhanced growth of HRCs with the application of low concentration of 2,4-D followed by IAA was noted. Zhao et al. [82] reported that with the addition of yeast polysaccharide elicitor (YPS) to HRCs, rutin and quercetin content increased two times when compared with the control. Further experiments resulted in a three-fold increase om flavonols, when YPS treatment phenylpropanoid pathway stimulation was combined with the process of medium renewal. As YPSs are commercially available, this research demonstrated an enhanced way for phenolic compound synthesis in HRCs [82]. Ethephon is a PGR that releases ethylene which is subsequently absorbed by plants. It results in enhanced flowering, fruit ripening and secondary metabolite production [102]. Li et al. [72] tested the effect of ethephon on HRCs of Tartary buckwheat and noted enhancement in anthocyanin biosynthesis when supplemented with a concentration of 0.5 mg/L. This led them to conclude that the biosynthesis of anthocyanin plays a crucial part in the response of buckwheat to ethephon-induced stress [72].

Research into gene transformation in Tartary buckwheat focused on transcription factors (TFs) of the MYB family, which are common in plants [103]. Jasmonites (JAs) are plant hormones known to induce the biosynthesis of different secondary metabolites. Transcriptional repressors, jasmonate ZIM domain (JAZ) proteins interact with different TFs that have various roles in regulating JA-responsive expression of genes [104]. For example, genes which encode JAZ are the integral factor in the anthocyanin synthesis as a response to a certain type of stress [103]. Another one of the types that takes part in JA-induced biosynthesis of secondary metabolites is MYB type. R2R3-MYB TFs are known to play a substantial part in regulating the biosynthesis of secondary metabolites [105]. To examine the characteristics of MYB TFs and their derivatives in planta, Zhou et al. [83] investigated rutin accumulation in HRCs of Tartary buckwheat. A previously unknown R2R3-MYB TF FtMYB11 was isolated and classified as a subgroup 4 of R2R3-MYB TFs. It was also found that the level of FtMYB11 expression was substantially induced by Jas, which led to the conclusion that FtMYB11 is a JA-responsive TF and its activity might be regulated by JAZ repressors. Analysis of rutin biosynthesis genes in HRCs qRT-PCR revealed that FtMYB11 repressed the expression of most of the key enzyme genes, which led to the conclusion that FtMYB11 is a master regulator repressing rutin biosynthesis [106]. A subsequent study of HRCs described a clade of JA-responsive MYB repressors (FtMYB13, FtMYB14, FtMYB15, and FtMYB16) and their key involvement in repressing phenylpropanoid biosynthesis. A study that focused on FtMYB16 interactions with Ftimportin-α1 (FtPinG0006805200) and subsequent regulation of rutin biosynthesis concluded that FtMYB16 act as a repressor in both root growth and rutin accumulation [107]. Moreover, Ftimportin-α1 directly regulates the import of FtMYB16 to the nucleus and acts as a promoter of FtMYB16 transcriptional activity on its target genes [108].

### 2.4. Transgenic Buckwheat Plants

Genetic plant transformation has become one of the highly promising tools in plant breeding and research [73]. It has been previously engaged in defining plants’ genes functions [109]. Although an efficient protocol for *A. tumefaciens* for common buckwheat was developed by Neskovic et al. [93] who established HRCs with *A. rhizogenes*, limited research regarding the subject followed in subsequent years. Miljus-Djukic et al. [110] studied the efficiency of various strains of *A. tumefaciens* and their experiments showed that the A281 strain exhibited stronger virulence than the other strains (Ach5 and A6). The same authors genetically transformed buckwheat plants using *A. tumefaciens* A281 strain as vectors. Incubated cotyledon fragments with A281 strain harbouring pGA472 were carrying the *neomycin phosphotransferase II (nptII)* gene responsible for kanamycin resistance. Regenerated seedlings exhibited the ratio of resistant to sensitive approximately three to one [110]. To test the efficiency of different strains in planta of *A. tumefaciens* (LBA4404 and pBI121) apical meristems of young buckwheat seedlings were inoculated. The results showed the transformation efficiency of 36% and 70% with strain LBA4404 and pBI121 strain, respectively [109]. Transgenic plants of common buckwheat were also obtained from infecting hypocotyl fragments with *A. tumefaciens* LBA4404 strain harbouring vector pBI121 which contains genes of *neomycin phosphotransferase II (npt II)* and β-*glucuronidase (gus)* [56]. Moreover, these authors tested cotyledon and hypocotyl explants and, contrarily to Miljus-Djukic et al. [110], observed better efficiency of the latter for the production and recovery of regenerants [56]. These reports allowed for the improvement of protocols in buckwheat *Agrobacterium*-mediated genetic transformation.

HRCs have also been used in the attempts to improve gene transformation efficiency with the use of *A. tumefaciens* [111]. It has been shown to influence the production of certain phytohormones and subsequently enhanced proliferation and tumour formation [112]. Transformation of common buckwheat via the establishment of in vitro callus was achieved by using the *A. tumefaciens* LBA4404 strain, which contained pHZX1 binary vector. Transgenic plants that exhibited overexpression of *AtNHX1* were regenerated. *AtNHX1* is a vacuolar Na+/H+ antiporter gene from *Arabidopsis thaliana*. Overexpression of *AtNHX1* showed improved salt tolerance in other species: tomato [113], *Brassica napus* [114], rice [115], perennial ryegrass [116] and wheat [117]. In regenerated transgenic common buckwheat, line growth was less inhibited by salt stress when compared to wild type, which demonstrated that overexpressing Na+/H+ antiporter cDNA can have a positive effect on improved salt tolerance [118]. Deciphering metabolic pathways will in future provide molecular bases for metabolic engineering studies.

## 3. Future Perspectives

Buckwheat, due to its high content of micro- and macronutrients, as well as its health benefits and potential, was established as functional food and pharmaceutical plant. However, as the population and the environment are exposed to constant and rapid changes, combined with scientific progress, there are therefore several aspects of buckwheat research that have to be addressed. As described in this review, in vitro callus induction and plantlet regeneration of common and Tartary buckwheat is rather well researched. There are some challenges plant breeders are faced with. The main problem with common buckwheat is the short life of its single flower and a growing period which lasts from 70 to 90 days [119]. Indeterminate type of growth and flowering makes it difficult to determine harvesting time. Susceptibility of buckwheat to biotic stresses such as ground frost, low water supply, drought and photoperiod causes flowering and embryo abortions. *F. homotropicum* has been cross-pollinated with common buckwheat and Tartary buckwheat in order to transfer genes that have a greater resistance to frost and a higher seed yield. These attempts, however, have been unsuccessful due to the barriers preventing cross-pollination between different species [120]. Common buckwheat plants are dimorphic with two types of flowers—Pin with pistils longer than stamens, and Thrum with pistils shorter than stamens—resulting in self-incompatibility [19,119]. Fertilisation occurs between both flower types after cross-pollination [121]. Tartary buckwheat is a homostylous species with flowers that contain anthers and stigmas of the same height. Among the most important reasons for the low yield are: self-incompatibility; insufficient fertilisation; embryo abortion; sensitivity to heat and drought stress; and assimilation deficiency that occurs in aging plants [122].

Protoplast fusion and subsequent in vitro plant regeneration, which leads to somatic hybridisation, offers opportunities for transferring entire genomes from one plant into another regardless of the interspecific crossing barriers [123]. Somatic hybridisation has been successfully used for a number of intra- and inter-specific, intergenic, inter-tribal and even inter-familial combinations [124,125]. The literature data about the protoplast fusion of the buckwheat species are limited. There was only one success with obtaining hybrid calli of common buckwheat (+) Tartary buckwheat achieved by Lachmann et al. [126] using polyethylene glycol (PEG)-mediated protoplast fusion. Although the authors highlighted the hybrid nature of obtained calli, the possibility of plant regeneration was not shown. Currently, the most promising and efficient technique is protoplast electrofusion by staining the protoplasts with different fluorescent colours, enabling the hybrid cells to be selected at the beginning of the experiments.

Draft genome sequences for common and Tartary buckwheat will help in gaining understanding of genetic mechanisms and regulation of specific traits, proteomic, transcriptomic, metabolomics and epigenomic approaches. For example, Penin et al. [127] characterised 1.5 Gb genome along with the reference assembly of common buckwheat. This will lead to opportunities to determine functions that will help in the population improvement programmes as well as to examine their mechanisms. These authors used the draft genome in order to reference it with genotyping-by-sequencing (GBS) analysis and discover the location of the 5.4 Mbp S-allelic region along with candidate genes responsible for controlling buckwheat heteromorphic self-incompatibility [127].

Genome editing with the RNA-guided clustered regularly interspaced short palindromic repeats-associated protein 9 (CRISPR/Cas9) technology is emerging as a valuable tool in research on plant functional genomics [128]. It has the potential to revolutionise the modification of traits and characteristics in plants by functional gain or loss via insertion of single-guide RNA (sgRNA) into the plant genome [129]. CRISPR/Cas9 is far more efficient, simpler and gives the possibility to edit multiple target genes concurrently, compared with zinc finger nucleases (ZFNs) and transcription activator-like effector nucleases (TALENs) [130]. Developing efficient protocol for buckwheat will help to generate plant varieties with improved traits such as better resistance to biotic and abiotic stresses and improved breeding timelines. CRISPR/Cas9 gene editing technology was successfully used in *Brachypodium* [131], *Arabidopsis thaliana*, sorghum [132], rice [133], barley [134], wheat [135] and corn [136], but not in *Fagopyrum* species. The CRISPR/Cas9 system can also be used in targeting microRNAs (miRNA) in order to gain insight into miRNA regulatory pathways. This has been performed in soybean [137] and in rice [138]. *Agrobacterium*-mediated genetic transformation, particle bombardment and PEG-transformation and protoplast fusion allowed us to obtain genome-modified lettuce [73], *Arabidopsis*, tobacco [139] and rice [140], apple [141], petunia [142]; however, no regenerants were obtained. The approach to standardize factors affecting genetic transformation in buckwheat by particle bombardment or via any of the direct-DNA delivery methods will help to excel the scarce research which mainly focused on optimising the method protocols.

To summarise, understanding the morphological, physiological and molecular parameters that affect the development of buckwheat plants will be relevant for revealing the complex mechanisms. This in turn will have an impact on successfully modulating yield productivity and stability for future buckwheat breeding programmes.

## Figures and Tables

**Table 1:** Optimal conditions for the callus induction from different explants in *F. esculentum* and *F. tataricum*.

Species	Explant	Basal Medium	PGRs	References
*F. esculentum*	Hypocotyl and cotyledon	White’s medium	5.0–10.0 mg/L 2,4-D	[13]
	Hypocotyl	MS	2.0 mg/L 2,4-D + 0.1–2.0 mg/L 6-BA	[14]
	Hypocotyl	B5	1.0–5.0 mg/L 2,4-D + 0.05–2.0 mg/L 6-BA	[14]
	Immature inflorescence	B5	1.0 mg/L 2,4-D + 2.0 mg/L NAA	[17]
	Hypocotyl derived protoplast	MS	2.0 mg/L NAA + 1.0mg/L 6-BA	[19]
	Leaf and stem	MS	1.0 mg/L 2,4-D	[16]
	Hypocotyl and cotyledon	MS	2.0 mg/L 2,4-D + 0.5 mg/L 6-BA	[38]
	Leaf	MS	1.0 mg/L 2,4-D	[39]
	Cotyledon	MS	2.0 mg/L 2,4-D + 0.2 mg/L KT	[40]
	Leaf	MS	2.0 mg/L 2,4-D + 0.2 mg/L KT	[41]
	Hypocotyl	MS	2.0 mg/L 2,4-D + 1.0 mg/L 6-BA	[42]
	Hypocotyl	MS	1.0–2.0 mg/L 2,4-D + 1.5 mg/L 6-BA	[43]
	Anther	B5	1.0 mg/LNAA + 2.0 mg/I 6-BA	[18]
	Anther	MS	2.0 mg/L KT + 2.0 mg/L 6-BA	[44]
*F. tataricum*	Immature embryos	B5	2.0 mg/L thiamine + 1.0 mg/L pyrioxidine + 1.0 mg/L nicotinic acid + 2000 mg/L casein hydrolysate + 2.0 mg/L 2,4-D + 0.5 mg/L IAA + 0.5 mg/L NAA + 0.2mg/L KT	[34]
	Hypocotyl derived protoplast	MS	1.0 mg/L NAA + 1.0 mg/L 6-BA	[20]
	Hypocotyl and cotyledon	MS	2.0 mg/L 2,4-D + 1.0 mg/L KT	[45]
	Hypocotyl	MS	4.0 mg/L 2,4-D + 1.0 mg/L 6-BA	[46]
	Hypocotyl	MS	3.5 mg/L 2,4-D + 0.8 mg/L 6-BA	[37]

## Data Availability

Not Applicable.
